# Feeling Voices

**DOI:** 10.1371/journal.pone.0053585

**Published:** 2013-01-16

**Authors:** Paolo Ammirante, Frank A. Russo, Arla Good, Deborah I. Fels

**Affiliations:** 1 Department of Psychology, Ryerson University, Toronto, Canada; 2 Centre for Learning Technologies, Ryerson University, Toronto, Canada; University of Montreal, Canada

## Abstract

Two experiments investigated deaf individuals' ability to discriminate between same-sex talkers based on vibrotactile stimulation alone. Nineteen participants made same/different judgments on pairs of utterances presented to the lower back through voice coils embedded in a conforming chair. Discrimination of stimuli matched for F0, duration, and perceived magnitude was successful for pairs of spoken sentences in Experiment 1 (median percent correct = 83%) and pairs of vowel utterances in Experiment 2 (median percent correct = 75%). Greater difference in spectral tilt between “different” pairs strongly predicted their discriminability in both experiments. The current findings support the hypothesis that discrimination of complex vibrotactile stimuli involves the cortical integration of spectral information filtered through frequency-tuned skin receptors.

## Introduction

The investigation of haptic speech perception has a long history. In 1924, for example, Gault [1] trained an artificially-deafened subject to identify thirty-four spoken words presented to his palm through a tube. The Tadoma method, developed around the same time to facilitate speech perception in deafblind individuals, involves placing thumb and fingers on a talker's lips and jawline, respectively [2]. In the 1980s, Brooks and colleagues investigated speech perception using tactile vocoders that filter an acoustic waveform and transduce it into vibratory patterns that are felt on the skin [3]–[5]. Using this apparatus, they trained an individual to acquire a 250-word vocabulary [5]. Correct identification of the number of syllables and stress patterns of incorrectly identified words suggest the haptic sense was used to track the amplitude envelope of speech as it unfolds over time. Here we evaluate vibrotactile sensitivity to spectral information contained in speech.

Our previous study on vibrotactile discrimination of musical timbre [6] is, to our knowledge, the only published study to directly investigate vibrotactile sensitivity to spectral information. Tones produced by a musical instrument and voiced sounds produced by the vocal cords are complex periodic waveforms. Component frequencies of such waveforms include the *fundamental frequency (F0)*, which is usually associated with the perceived pitch of a musical tone, and *harmonics* at integer multiples of F0. The resonance properties of a musical instrument or the vocal tract give rise to frequency bands of higher amplitude called *formants* that boost those harmonics falling within it. The timbres of different musical instruments or different voices producing the same sound (e.g., a tone at middle C [262 Hz] or a vowel at 220 Hz) are differentiated in part by the relative amplitudes of F0 and its harmonics, i.e., frequency spectrum. We found that artificially-deafened individuals as well as a sample of individuals from the deaf and hard-of-hearing (DHH) community readily discriminated by touch alone piano, cello, and trombone tones matched for F0, duration, and perceived magnitude, and synthesized tones that differed only in spectral content.

In the current study, DHH individuals were recruited to investigate vibrotactile discrimination of identical sentence (Experiment 1) and vowel utterances (Experiment 2) from same-sex talkers matched for F0, duration, and perceived magnitude. On the one hand, based on our previous findings, we expect that spectral differences between same-sex talkers, i.e., inter-individual differences in formant frequencies and/or their relative amplitudes, should lead to vibrotactile discrimination.

On the other hand, there are at least two reasons to anticipate difficulties with the task. First, whereas the timbres of musical instruments vary greatly according to the unique resonance properties of the materials with which they are constructed, inter-individual differences in vocal timbre, being governed primarily by modest differences in vocal tract morphology, are less pronounced [7]. Second, whereas in our previous study [6], variation in amplitude and spectrum over the course of each 2 s stimulus was either highly constrained (musical instrument samples) or absent (synthesized tones), the current 2 s speech stimuli are characterized by numerous rapid changes. These include transient changes in amplitude, such as at syllable onsets in sentences, and spectro-temporal change, such as formant transitions occurring during the articulation of vowel sounds. These dynamic changes might obscure spectrum and lead to poor discrimination.

## Experiment 1

### Methods

#### Ethics Statement

The study was approved by the Ryerson Ethics Board (REB) at Ryerson University and was conducted according to their human subject guidelines. Participation in the study was agreed to in writing by signing an REB-approved consent form.

#### Participants

Nineteen individuals (9 females) aged 23–64 (M = 42.1; SD = 12.9) were recruited from Toronto's deaf community. All had participated in a previous study [6]. Participants were compensated $20.

#### Apparatus

Complex vibrotactile waveforms were driven by an acoustic signal and presented to the back via a pair of voice coils embedded in a conforming chair [8], [9]. The voice coils were 1 inch in diameter and made contact with the left and right sides of the lumbar region of the back.

Eleven participants reported some hearing at high intensity and five of these participants wore hearing aids. To eliminate any possibility of auditory stimulation, all participants wore sound attenuating earmuffs with a noise reduction rating of 26 dB, and those with hearing aids were asked to turn their devices off for the duration of the experiment.

#### Stimuli & Procedure

Stimuli were six recorded utterances of the sentence “Can you tell who is singing this /ei/?”: one utterance from each of three different female talkers and one from each of three different male talkers. Recordings were made at a sampling rate of 44.1 kHz using a Rode NTK microphone. Talkers attempted to match their utterances in duration and F0 to a 2 s standard tone presented at F0 of 220 Hz for female talkers and 110 Hz for male talkers. Since F0 continuously varies in speech (and is thus unlikely to act as a stable perceptual cue to participants), the standard tone served to broadly center F0 and discourage gross deviations in range. All utterances were shorter than the target duration (M = 1.72 s; SD = .09); the maximum difference in duration between utterances was 12%. The maximum difference in F0 semi-interquartile range between utterances did not exceed 1 semitone. All stimuli were also equated for perceived magnitude of vibration. Average ratings were taken from three normal hearing judges who iteratively adjusted the magnitude of a target stimulus until it was perceived to match a standard. Judges were artificially deafened to the sound output of the voice coils by white noise presented over headphones to mask air-conducted sound and a vibrotactile stimulus applied to the mastoid bone to mask bone-conducted sound [6].

Participants made same/different judgments for stimulus pairs presented with an inter-stimulus interval of 1 s. A practice block of 5 trials with feedback was followed by 2 experimental blocks of trials without feedback. Only the latter were entered into analysis. Each experimental block presented pairs from either the three female or three male talkers, and the order of presentation of the blocks was randomized. Within blocks, all possible talker pairs were presented once (3 talkers squared = 9 trials). Thus, one-third of the pairs were same and two-thirds were different. A professional sign language interpreter delivered instructions to participants using American Sign Language.

### Results

Separate two-tailed binomial tests were performed on each participant's responses across all stimuli, and revealed percent correct to be significantly above chance (*p*<.05) in 14 of 19 participants, Mdn = 83.33%. (d' values obtained from a signal detection analysis [10] indicated that responses in both experiments were unbiased: Experiment 1 [Mdn = 2.54]; Experiment 2 [Mdn = 2.35].)

**Figure 1:**
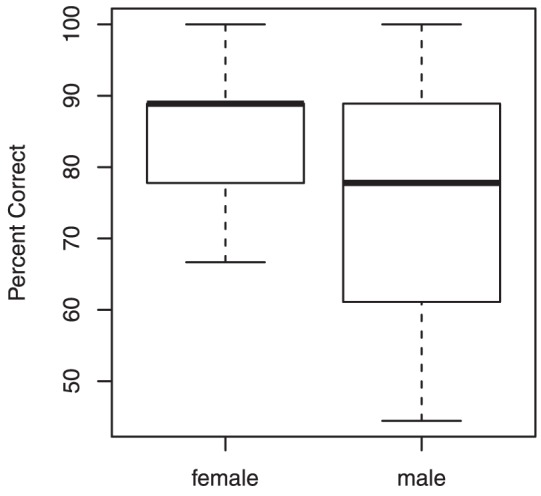
Boxplots of percent correct by participant across two conditions. Median is shown as a bolded line. Lower and upper edges of the boxes indicate lower and upper quartiles, respectively, and whiskers indicate sample minima and maxima.

As shown in Figure 1, percent correct was higher for female talkers than male talkers, but Friedman's ANOVA revealed no significant effect of sex, *F*(1) = 2.57, *p*<.11.

Table 1 shows percent correct by stimulus. As there were no effects of order of presentation for “different” pairs, percent correct was collapsed across complementary pairs (e.g., “Male-B/Male-C” and “Male-C/Male-B”). Binomial tests revealed percent correct to be significantly above chance (*p*<.05) for all stimulus pairs except Female-A/Female-B.

**Table 1:** Percent correct by stimulus based on 19 responses for “same” sentences, and on 38 responses collapsed across complementary “different” pairs.

	Female-A	Female-B	Female-C		Male-A	Male-B	Male-C
Female-A	95*			Male-A	100*		
Female-B	45	95*		Male-B	66*	89*	
Female-C	97*	100*	95*	Male-C	74*	66*	74*

* *p*<.05.

## Experiment 2

### Methods

#### Participants

The same 19 individuals completed Experiment 2 on the same day as Experiment 1.

#### Apparatus

The apparatus was identical to Experiment 1.

#### Stimuli & Procedure

Stimuli were six recorded utterances of the dipthong /ei/ made by the same three female and three male talkers as in Experiment 1. Talkers attempted to match their utterances in F0 to a standard tone presented at either low or high pitch. For females, low pitch was 220 Hz and high pitch was 440 Hz; for males, low pitch was 120 Hz and high pitch was 220 Hz. In all utterances, F0 minimally deviated from these targets (M = 22 cents; SD = 17.2). The central 2 s portion of each vowel utterance was extracted using audio editing software. All stimuli were equated for perceived magnitude using the same protocol as Experiment 1.

Procedures used were identical to Experiment 1, but there were 4 blocks of experimental trials: Female/low pitch, Female/high pitch, Male/low pitch, and Male/high pitch. The order of presentation of both trials within blocks and the blocks themselves was randomized. Within blocks, all possible talker pairs were presented once. Each block presented pairs from either the three female or three male talkers, for a total of 36 trials.

### Results

Separate two-tailed binomial tests on each participant's responses across conditions showed percent correct to be significantly above chance (*p*<.05) in 17 of 19 participants, Mdn = 75%. Neither of the two participants scoring at chance in Experiment 2 scored at chance in Experiment 1.

**Figure 2:**
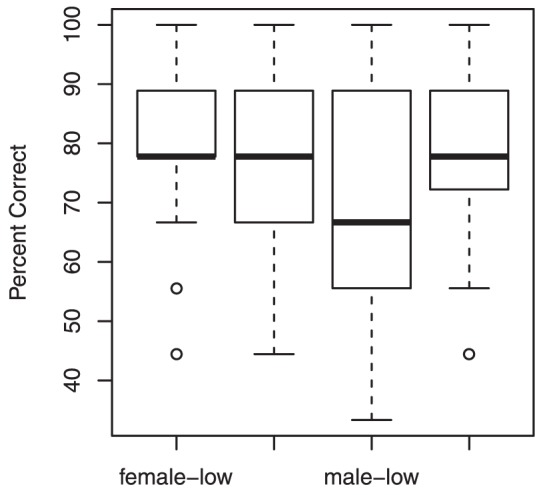
Boxplots of percent correct by participant across four conditions. Outliers are shown as circles.

As shown in Figure 2 and consistent with a trend observed in Experiment 1, percent correct was lower for low-pitched male talkers, but Friedman's ANOVA revealed no significant difference in percent correct between conditions, *F*(3) = 1.28, *p*<.74.

Table 2 shows percent correct by stimulus after collapsing across complementary “different” pairs and across low- and high-pitched vowels within sex. Binomial tests showed percent correct to be significantly above chance (*p*<.05) for 10 of 12 talker pairs. Interestingly, as in Experiment 1, percent correct was at chance for Female-A/Female-B. Moreover, percent correct for corresponding talker pairs in Experiments 1 and 2 was significantly correlated, *r*(10) = .79, *p*<.01. Taken together, these data suggest participants relied on global cues common to spoken sentences and sung vowels rather than local timing differences between talker pairs, such as the timing of syllable onsets, available in sentences but not vowels.

**Table 2:** Percent correct by stimulus based on 38 responses for “same” vowels, and on 76 responses collapsed across complementary “different” pairs.

	Female-A	Female-B	Female-C		Male-A	Male-B	Male-C
Female-A	92*			Male-A	95*		
Female-B	54	84*		Male-B	70*	79*	
Female-C	84*	90*	90*	Male-C	55	79*	90*

* *p*<.05.

#### Acoustic Analysis

An acoustic analysis of the stimuli was conducted to test the hypothesis that, for both spoken sentences and sung vowels, larger global differences between “different” talker pairs should result in those pairs being more discriminable. In order to gain more statistical power, data from both orders of presentation were included for each pair. Root-mean-square voltage was first measured to verify that global differences in intensity were not driving discriminability between talkers. This is a measure of overall energy in the signal irrespective of frequency content. Percent correct was not significantly correlated with the absolute difference in root-mean-square voltage between stimuli either for the 12 “different” spoken sentences pairs, *r*(10) = .36, *p*<.25, or the 24 “different” vowel pairs, *r*(22) = .14, *p*<.53.

**Figure 3:**
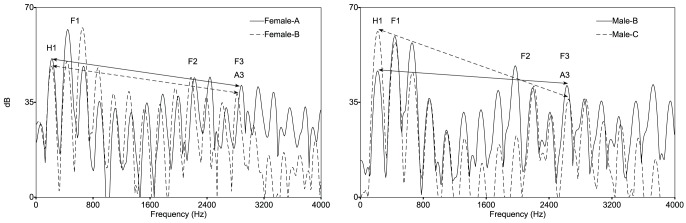
Spectral slices (50 msec in length, starting from 1 s) of 220 Hz vowel utterances. F0 and its harmonics are sharp peaks at 220 Hz and integer multiples. Formants (F1, F2, F3) can be seen as shallower peaks each containing multiple harmonics. (left panel) Where spectral tilt (H1-A3) was nearly identical between Female-A and Female-B, responses were at chance (40% correct); (right panel) difference in spectral tilt between Male-B and Male-C was large and elicited 87% correct responses.

Next, a global spectral measure was used to investigate whether such information may have guided discrimination of both spoken sentences and sung vowels. *Spectral tilt* refers to the reduction of the high frequency spectrum relative to the low frequency spectrum. As shown in Figure 3, spectral tilt was measured here as H1-A3, or as the difference (in dB) between the amplitude of the first harmonic (H1) and the amplitude of the most prominent harmonic in the third formant (F3 = formant; A3 = harmonic) [11], [12]. The acoustic correlate of H1-A3 is breathiness; a breathy voice with stronger H1 has a large spectral tilt, while a creaky voice with more energy at A3 has a small spectral tilt [13].

Spectral tilt was estimated using the acoustic analysis software Praat [14] for the 18 recordings used as stimuli from Experiments 1 and 2. For Experiment 1, the voiced portions of the sentences were extracted for further analysis (mean duration after extraction = 1198 msec); for Experiment 2, the entire 2 s vowel utterance was used. For each recording, after downsampling to 16 kHz, F3 was estimated using linear prediction at regular temporal intervals. Next, the long-term average spectrum was calculated using a 100 Hz bandwidth at each of 12 equally-spaced intervals (i.e., ∼100 msec) for sentences and at each of 20 equally-spaced intervals (i.e., 100 msec) for vowels. For each interval, H1 and A3 were identified in the frequency spectrum as the maximum amplitude peaks within 10% of the frequencies of F0 and F3, respectively, and A3 was subtracted from H1 [15]. Global estimates of spectral tilt for each stimulus were obtained by averaging these values across intervals. Finally, for each “different” pair, the absolute difference in global spectral tilt between stimuli was obtained.

**Figure 4:**
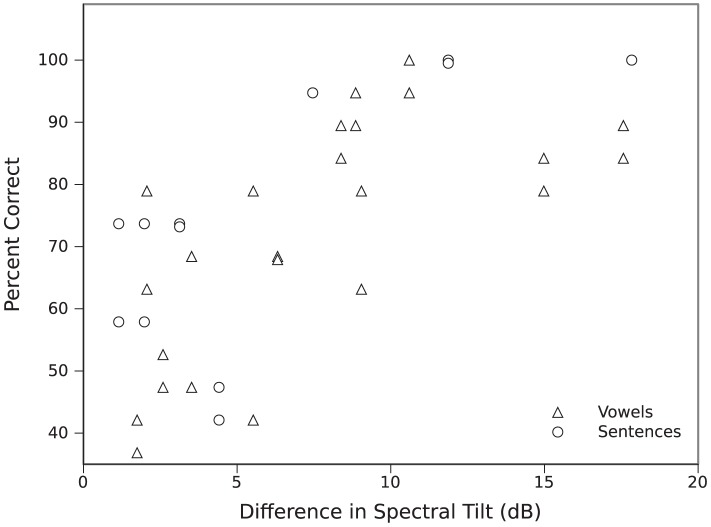
Correlation between the absolute difference in global spectral tilt and percent correct.

Percent correct was correlated with the absolute difference in global spectral tilt for “different” pairs. Significant correlations were observed for the sentences in Experiment 1, *r*(10) = .73, *p*<.01, for the vowels in Experiment 2, *r*(22) = .68, *p*<.001, and across both experiments, *r*(34) = .67, *p*<.0001 (as shown in Figure 4). These findings suggest that participants used global spectral cues available in both spoken sentences and sung vowels to discriminate talker pairs.

## Discussion

Two experiments investigated deaf individuals' ability to discriminate between same-sex talkers based on vibrotactile stimulation alone. Nineteen participants made same/different judgments on pairs of utterances presented to the lower back through voice coils embedded in a conforming chair. Discrimination of stimuli matched for F0, duration, and perceived magnitude was successful for pairs of spoken sentences in Experiment 1 (median percent correct = 83%) and pairs of vowel utterances in Experiment 2 (median percent correct = 75%). The finding that discrimination was correlated for sentences and vowels suggests that participants were largely insensitive to local transient changes in amplitude, such as syllable onset available in spoken sentences, and spectro-temporal changes, such as formant transitions occurring in the talkers' articulation of vowel sounds. Moreover, analysis of global spectrum averaged across each utterance showed greater absolute difference in spectral tilt between stimuli in “different” pairs to be a strong predictor of their discriminability for both sentences and vowels. Taken together, these data suggest participants relied on more stable spectral cues available in both sets of stimuli.

How does vibrotactile sensitivity to spectral information arise? In audition, the tonotopic organization of frequency-tuned cells filters complex sounds into their component frequencies in critical bands of about one-third of an octave. Timbral discrimination is thought to follow from the cortical integration of the relative amplitudes of these signals in a process called profile analysis [16].

Given that the identical mechanical energy gives rise to auditory and vibrotactile sensations of sound by bending and distorting cells in the ear and skin, respectively, it seems reasonable that information from a haptic filterbank can likewise be cortically integrated. Indeed, at least four types of frequency-tuned skin receptors are recognized [17], [18]. For example, Pacinian corpuscles have peak sensitivity to vibration between 225 and 275 Hz and are found primarily within the dermis, while Meissner's corpuscles are most sensitive to vibration below 50 Hz and are found just below the epidermis. Evidence of a critical band function comes from studies showing that, as with auditory perception, perceived magnitude of pairs of pure tones presented to the skin either successively [19] or simultaneously [20] is summed only when the frequencies of the tones are widely-spaced.

In the current study, we demonstrate in a sample of DHH individuals the viability of the haptic sense for the discrimination of same-sex talkers. Talker identification is a disorienting problem that DHH individuals regularly face in vocational [21] and entertainment [22] settings. The current findings suggest a valuable role for vibrotactile information that may be used to supplement assistive listening devices used by DHH individuals.
